# Phenol-Rich *Feijoa sellowiana* (Pineapple Guava) Extracts Protect Human Red Blood Cells from Mercury-Induced Cellular Toxicity

**DOI:** 10.3390/antiox8070220

**Published:** 2019-07-11

**Authors:** Fabiana Tortora, Rosaria Notariale, Viviana Maresca, Katrina Vanessa Good, Sergio Sorbo, Adriana Basile, Marina Piscopo, Caterina Manna

**Affiliations:** 1 Department of Precision Medicine, School of Medicine, University of Campania “Luigi Vanvitelli”, via Luigi de Crecchio, 80138 Naples, Italy; 2 Department of Biology, University of Naples Federico II, via Cupa Nuova Cinthia, 80126 Naples, Italy; 3 Department of Biochemistry & Microbiology, University of Victoria, Victoria, BC V8W 3R4, Canada; 4 Ce.S.M.A, Microscopy Section, University of Naples Federico II, via Cupa Nuova Cinthia, 80126 Naples, Italy

**Keywords:** feijoa extracts, mercury, red blood cells, oxidative stress, glutathione, thiol groups, functional food

## Abstract

Plant polyphenols, with broadly known antioxidant properties, represent very effective agents against environmental oxidative stressors, including mercury. This heavy metal irreversibly binds thiol groups, sequestering endogenous antioxidants, such as glutathione. Increased incidence of food-derived mercury is cause for concern, given the many severe downstream effects, ranging from kidney to cardiovascular diseases. Therefore, the possible beneficial properties of *Feijoa sellowiana* against mercury toxicity were tested using intact human red blood cells (RBC) incubated in the presence of HgCl_2_. Here, we show that phenol-rich (10–200 µg/mL) extracts from the *Feijoa sellowiana* fruit potently protect against mercury-induced toxicity and oxidative stress. Peel and pulp extracts are both able to counteract the oxidative stress and thiol decrease induced in RBC by mercury treatment. Nonetheless, the peel extract had a greater protective effect compared to the pulp, although to a different extent for the different markers analyzed, which is at least partially due to the greater proportion and diversity of polyphenols in the peel. Furthermore, *Fejioa sellowiana* extracts also prevent mercury-induced morphological changes, which are known to enhance the pro-coagulant activity of these cells. These novel findings provide biochemical bases for the pharmacological use of *Fejioa sellowiana*-based functional foods in preventing and combating mercury-related illnesses.

## 1. Introduction

*Feijoa sellowiana (Feijoa)*, commonly known as pineapple guava, is an evergreen shrub in the Mytraceae family that is native to South America. It is commonly cultivated in tropical and subtropical countries, such as Brazil, Uruguay, Paraguay, and Argentina, but its cultivation has been extended to other countries, including Italy. *Feijoa* fruit, an intensely fragrant dark green oval berry, is commonly eaten fresh or as a variety of commercially available processed foods, such as jam, ice cream, and yoghurt [1,2].

The advances in chemical composition and biological activities of different botanical parts of *Feijoa* have recently been summarized in a mini-review by Fan Zhu [3]. The fruit is the most utilized botanical part of *Feijoa*, and its nutritional value is generally defined by the presence of dietary fiber, essential amino acids, potassium, and vitamins, including vitamin C. Recent studies have added to *Feijoa* nutritional properties, including high folic acid content, and particularly high iodine (3 mg/100 g wet weight) [4]. This fruit also contains pharmacologically relevant bioactive phytochemicals in the pulp and peel, including polyphenols, the major contributors of its bioactivities [3]. Plant polyphenols are widely known for their antioxidant activity [5], and *Feijoa* contains a variety of these compounds, including epigallocatechin, procyanidin B2, epigallocatechin gallate, myricetin-3-O-galactoside, epicatechin gallate, quercetin-3-galactoside, and quercetin-3-rhamnoside [6]. *Feijoa* extract has demonstrated antioxidant activity in a variety of human blood cells, as well as in vivo in rats [7,8]. Potential mechanisms of this activity have been studied, first by Rossi et al., wherein *Feijoa* extract suppressed nitric oxide production in murine macrophages via either attenuation of the nuclear factor κB (NF-κB) or activation of mitogen-activated protein kinase (MAPK) [9]. Additional activities include anti-microbial, anti-inflammatory, anti-cancer, and anti-diabetic capacities [10,11,12,13,14,15]. In human intestinal epithelial cells, *Feijoa* fruit improved lactase and sucrose-isomaltase activity and reduced proliferation, but was not cytotoxic, and also prevented lipid peroxidation [16].

The claimed *Feijoa* biological activities suggest that its bioactive components have great potential to be developed into functional food or natural supplements [17]. In addition to diet, *Feijoa* fruit extract is used for industrial purposes, including cosmetic preparation, due to its emollient, elasticizing, and dermo-purifying properties. Furthermore, *Feijoa* extracts have recently been considered as a potential sunscreen resource, due to their proven high absorption in the UV region [18].

Heavy metals are common environmental pollutants and come from natural and anthropogenic sources [19,20,21]. In recent decades, their contamination has increased dramatically [22,23,24]. In this respect, attention has focused on how bioactive phytochemicals may counteract heavy metal-induced body burden [25]. Of particular concern is the heavy metal mercury (Hg), which exists in several redox forms, each endowed with different bioavailabilities and toxicities [19,20]. It reacts adversely within organisms, but also remains and bioaccumulates within aquatic animal tissue, which is the major Hg source to humans. Hg poisoning in humans manifests as neurological, kidney, immune, and respiratory disorders, and is especially toxic in pregnant women [26,27,28,29,30]. Evidence from human and animal studies suggests that mercury also affects reproductive function [31], as do other heavy metals, such as copper [32,33,34] and cadmium [35]. Moreover, high levels of Hg may induce or exacerbate cardiovascular diseases [36,37,38]. Hg travels through the bloodstream and binds with high affinity to sulphydryl groups (-SH) [39]. This effectively increases the concentration of reactive oxygen species (ROS) in blood and tissue cells by inactivating or sequestering antioxidants, such as glutathione (GSH) [40]. Recent data from our group showed that hydroxytyrosol, an antioxidant phenol present in high concentrations in virgin olive oil, has the potential to modulate Hg toxicity in red blood cells (RBC), confirming the viability of designing nutritional strategies to counteract the adverse effects of Hg exposure in humans [41,42,43].

The aim of this study was to extend the relatively new research on the beneficial properties of *Feijoa* by testing the role of its components against Hg toxicity using intact human RBC. These anucleated cells, free of cellular organelles, represent a particularly important cellular model to study heavy metal toxicity [44,45]. First, we evaluate the ability of *Feijoa* peel and pulp acetonic extracts to protect human RBC from Hg-induced hemolysis. Then, the antioxidant properties of *Feijoa* extracts are measured in RBC by their ability to counteract the Hg-induced increase in ROS cellular generation. Protection against GSH, as well as membrane thiol depletion, are also measured. Moreover, confocal laser scanning microscopy was used to determine the protective effect of *Feijoa* extracts on Hg-treated RBC morphological alterations, as well as microvesicle (MV) formation. Exposure, even at low Hg concentrations, induces morphological changes that cause an increase in the procoagulant activity of these cells [46].

## 2. Materials and Methods

### 2.1. Chemicals

DCFH-DA (2,7-Dichlorodihydrofluorescein diacetate), mercuric chloride (HgCl_2_), and DTNB (5,5-dithiobis (2-nitrobenzoic acid), or Ellman’s reagent) were from Sigma Chemical Co. (St. Louis, Missouri, USA). Anti-Glicophorin A antibody (FITC) was purchased from antibodies-online.com (ABIN6253946, antibodies-online GmbH, Aachen, Germany).

### 2.2. Pulp and Peel Acetonic Extraction of Feijoa Fruits

A total of 60 g of fruit, subdivided into pulp and peel, either fresh or after storage at −5 °C, were treated with 0.8% water solution of Triton X-100 to remove epiphytic hosts normally found on the surface. They were then extensively washed in tap and distilled water and dried on filter paper. Peel and pulp separately underwent extraction with acetone for 15 min in a liquefier blender until homogenized. The resulting homogenate was centrifuged at 1800× *g* for 10 min; the supernatant was filtered, and the solvent was evaporated under reduced pressure at 45 °C for about 24 h. The dry extracts were solubilized with pure dimethyl sulfoxide (DMSO).

### 2.3. Preparation of Red Blood Cells and Treatment with HgCl_2_

Whole blood was obtained with informed consent from healthy volunteers at Campania University “Luigi Vanvitelli” in Naples, Italy. It was deprived of leucocytes and platelets by filtration in a nylon net and washed twice with isotonic saline solution (0.9% NaCl); the resulting intact RBC were resuspended in buffer A (5 mM Tris-HCl pH 7.4, 0.9% NaCl, 1 mM MgCl_2_, and 2.8 mM glucose) to obtain a 10% hematocrit, as previously described [41]. Intact RBC were incubated at 37 °C with 40 μM HgCl_2_ for 4 h or 24 h. For experiments with *Feijoa* pulp and peel extracts, stock solutions, prepared in DMSO as above described, were diluted in buffer A to a final DMSO concentration of about 0.02%, in order to avoid DMSO cytotoxicity. As a control, the effect of this volume of DMSO on RBC was evaluated and found to be negligible (data not shown). RBC from each donor were used for a single assay in triplicate. Each experiment was repeated on RBC obtained from three different donors.

### 2.4. Hemolysis Assay

RBC hemolysis extent was determined spectrophotometrically, according to Tagliafierro et al. [41]. After simultaneous treatment with HgCl_2_ and *Feijoa* extracts for 24 h, the reaction mixture was centrifuged at 1100× *g* for 5 min, and the released hemoglobin (Hb) in the supernatant was evaluated by measuring the absorption at 540 nm (*A*). As a positive control, packed RBC were used hemolyzed with ice-cold distilled water at 40:1 *v*/*v*, and by measuring the A540 of the supernatant obtained centrifuging the suspension at 1500× *g* for 10 min (*B*). The percentage of hemolysis was calculated as the ratio of the readings (*A*/*B*) × 100%.

### 2.5. Determination of Reactive Oxygen Species

ROS generation was determined using the dichlofluorescein (DCF) assay, according to Tagliafierro et al. [41]. Using this method, 250 µL of intact RBC (hematocrit 10%) were incubated with the non-polar, non-fluorescent 2′,7′-dichlorodihydrofluorescin diacetate (DCFH-DA) at a final concentration of 10 μM for 15 min at 37 °C. After centrifuging at room temperature at 1200× *g* for 5 min, the supernatant was removed, and the hematocrit was re-adjusted to 10% with buffer A. RBC were then treated concurrently with HgCl_2_ and *Feijoa* extracts in the dark for 4 h. After the incubation, 20 μL of RBC were diluted in 2 mL of water, and the fluorescence intensity of the oxidized derivative DCF was recorded (λ_exc_502; λ_em_520). The results were expressed as fluorescence intensity/mg of Hb.

### 2.6. Quantification of Intracellular Glutathione

Intracellular GSH content was determined spectrophotometrically by reacting with DTNB reagent, according to Van den Berg et al. [47]. After co-incubation with HgCl_2_ and *Feijoa* extracts for 4 h, the samples (0.25 mL) were centrifuged, and the cells were lysed by the addition of 0.6 mL of ice-cold water. Proteins were precipitated with 0.6 mL ice-cold metaphosphoric acid solution (1.67 g metaphosphoric acid, 0.2 g EDTA, and 30 g NaCl in 100 mL of water). After incubation at 4 °C for 5 min, the protein precipitate was removed by centrifugation at 18,000× *g* for 10 min, and 0.45 mL of the supernatant was mixed with an equal volume of 0.3 M Na_2_HPO_4_. Then, 100 μL of DTNB solution (20 mg DTNB plus 1% of sodium citrate in 100 mL of water) was then added to the sample, and after a 10 min incubation at room temperature, the absorbance of the sample was read against the blank at 412 nm.

### 2.7. Estimation of Free Sulfhydryl Groups in Isolated Red Blood Cell Membranes

Free sulfhydryl groups in membrane proteins (2.5 µg/µL for each sample estimated by Bradford assay) were assayed, according to the method of Ellman [48]. To do this, 650 µL of HgCl_2_-treated RBC were washed three times in 40 volumes of 5 mM sodium phosphate buffer pH 8.8, centrifuged at 10,000× *g* for 20 min at 4 °C, then washed several times with the same buffer, for the complete removal of Hb. Then, 1 mL of 0.1 M Tris–HCl pH 7.5 was added to 50 µL membrane protein. The colorimetric reaction was started by adding 50 µL of 10 mM DTNB in methanol. After 15 min of incubation at room temperature, the absorbance was read against the blank at 412 nm. Blanks were run for each sample in which DTNB was not added to methanol.

### 2.8. Morphological Analysis of Red Blood Cells

To investigate the possible protective effect of *Feijoa* extracts on alterations in the Hg-induced erythrocytes’ shape, we treated the cells both with 40 μM HgCl_2_ as well as 20 or 80 µg/mL of *Feijoa* peel and pulp extracts for 4h at 37 °C. After incubation, erythrocytes were washed twice with phosphate-buffered saline pH 7.4 (PBS), and counted in a Burker chamber. The confocal laser scanning microscope analyses were performed according to Nguyen [49], with few modifications. In brief, the cells were then fixed with 2% formaldehyde for 1 h at 4°C, then washed several times and incubated with anti-human anti glycophorin A FITC antibody for 30 min at 4°C in the dark. Afterwards, the samples were placed on glass slides and air-dried for 1 h. The slides were dipped quickly, and gently washed stepwise with ethanol from 50% to 75%, 90%, and then 100% for dehydration. Finally, cells were fixed in 2% formaldehyde and washed three times with PBS. For confocal laser scanning microscope imaging, several randomly selected frames from each sample were captured for morphological observation and statistical strength. Excitation and emission filters were set at 488 nm and 550–600 nm, respectively.

### 2.9. Statistical Analysis

Data were expressed as mean ± standard error of the mean (SEM). The significance of differences was determined by one-way ANOVA followed by a post hoc Tukey’s multiple comparisons test. GraphPad Prism 5 was utilized for statistical analysis.

## 3. Results

### 3.1. Feijoa Peel and Pulp Extracts Protect Against Hg-Induced Hemolysis.

**Figure 1:**
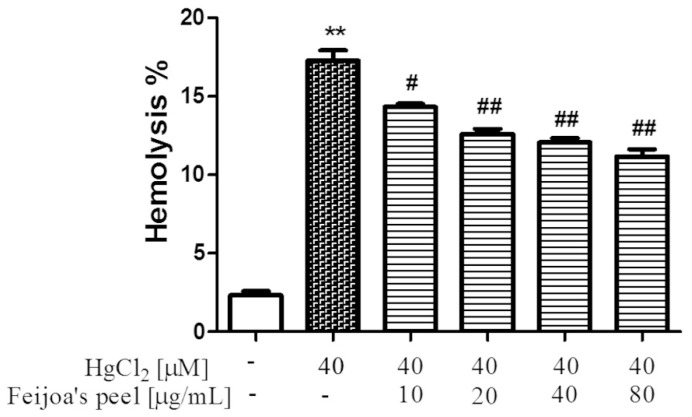
*Feijoa* peel acetonic extract reduces Hg-induced hemolysis. Cells were treated with 40 μM HgCl_2_ for 24 h with increasing acetonic extract concentrations. Data are the means ± standard errors of the mean (SEM) (*n* = 9). Statistical significance was calculated by one-way ANOVA followed by Tukey’s test. ** (*p* < 0.05) indicates a significant difference from cells lacking HgCl_2_ treatment. # (*p* < 0.05) and ## (*p* < 0.01) indicate significant differences from cells lacking *Feijoa* extract treatment.

**Figure 2:**
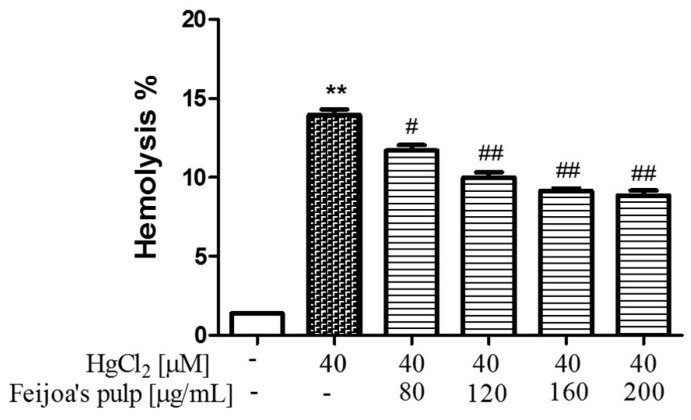
*Feijoa* pulp extract reduces Hg-induced hemolysis. Cells were treated with 40 μM HgCl_2_ for 24 h in the presence of increasing concentrations of extract. Data are the means ± SEM (*n* = 9). Statistical significance was calculated with one-way ANOVA followed by Tukey’s test. ** (*p* < 0.05) indicates a significant difference from cells lacking HgCl_2_ treatment. # (*p* < 0.05) and ## (*p* < 0.01) indicate significant differences from cells lacking *Feijoa* extract treatment.

The rescue of Hg-induced hemolysis by *Feijoa* extracts was assayed separately for peel and pulp, and the results are shown in Figure 1 and Figure 2, respectively. It can be seen that 24 h treatment of RBC with 40 µM HgCl_2_ resulted in approximately 13–17% hemolysis, compared to 1–2% in negative controls, as expected based on our previous work [41]. *Feijoa* peel extract potently reduced Hg-induced hemolysis compared to that of the pulp, with a significant 3% drop in hemolysis at 10 µg/mL, and a steady reduction of about 1% with each doubling of peel extract (Figure 1). *Feijoa* pulp treatment reduced cellular lysis in similar proportions, but the required protective extract concentration to do so was almost eight-fold greater than that of the peel extract (Figure 2). No cytotoxic effect was found by either *Feijoa* extracts up to the maximum concentration utilized in this study (data not shown).

### 3.2. Feijoa Peel and Pulp Extracts Reduce Reactive Oxygen Species Production in Red Blood Cells

**Figure 3:**
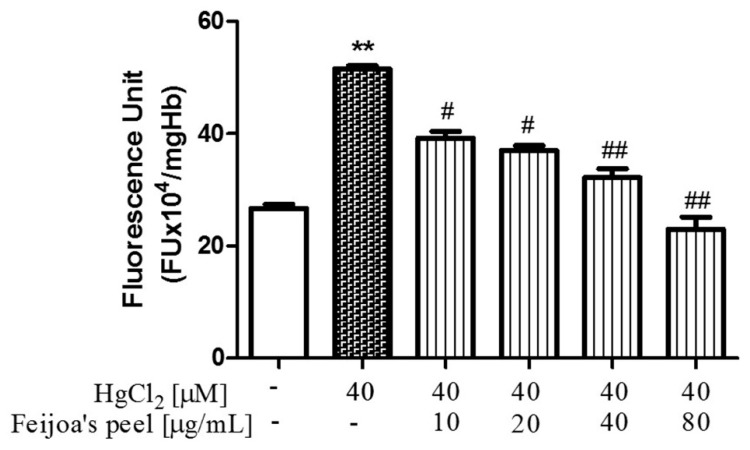
*Feijoa* peel extract protects against Hg-induced reactive oxygen species (ROS) production in red blood cells (RBC). Cells were treated with 40 μM HgCl_2_ for 4 h in the presence of increasing concentrations of extract. ROS production was determined by fluorescence unit means of the dichlofluorescein (DCF) probe. Data are the means ± SEM (*n* = 9). Statistical significance was calculated with one-way ANOVA followed by Tukey’s test. ** (*p* < 0.05) indicates a significant difference from cells lacking HgCl_2_ treatment. # (*p* < 0.05) and ## (*p* < 0.01) indicate significant differences from cells lacking *Feijoa* extract treatment.

**Figure 4:**
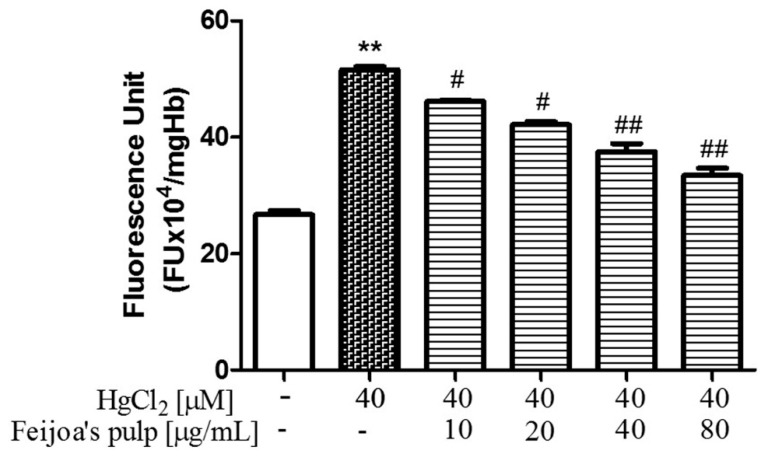
*Feijoa* pulp extract protects against Hg-induced ROS production in RBC. Cells were treated with 40 μM HgCl_2_ for 4 h in the presence of increasing concentrations of extract. ROS production was determined by fluorescence unit means of the DCF probe. Data are the means ± SEM (*n* = 9). Statistical significance was calculated with one-way ANOVA followed by Tukey’s test. ** (*p* < 0.05) indicates a significant difference from cells lacking HgCl_2_ treatment. # (*p* < 0.05) and ## (*p* < 0.01) indicate significant differences from cells lacking *Feijoa* extract treatment.

The fluorescence probe DCF assay elucidated the protective role of *Feijoa* extracts against oxidative stress in RBC, as reported in Figure 3 and Figure 4. ROS production increased nearly two-fold in Hg-treated RBC compared to the negative control. In contrast, co-incubation with 10, 20, 40, or 80 µg/mL of both peel and pulp acetonic extracts incrementally reduced ROS production in RBC. Similar to hemolysis, *Feijoa* peel extract prevented ROS production more potently than the pulp, and remarkably reduced fluorescence levels by approximately 50% at 10 µg/mL, to near control values at the highest extract concentration. At the same concentrations, *Feijoa* pulp extract also significantly reduced ROS production compared to the non-*Feijoa* protected RBC, reaching a maximum of about 50% at 80 µg/mL.

### 3.3. Peel and Pulp Extracts Prevent Hg-Induced Glutathione and Membrane Thiol Depletion in Red Blood Cells

**Figure 5:**
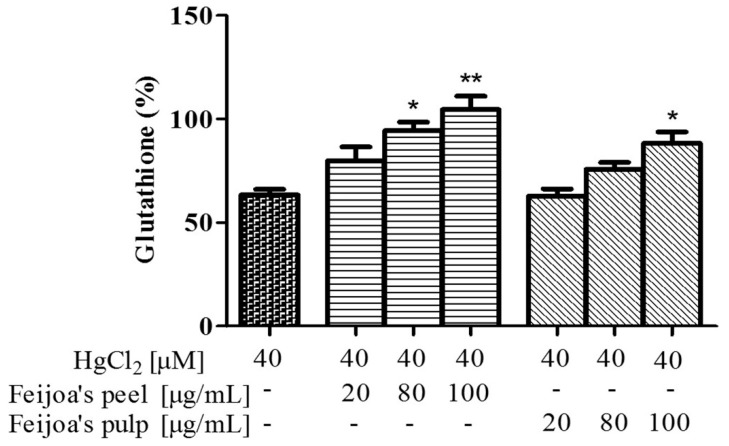
*Feijoa* peel and pulp extracts protect against Hg-induced glutathione (GSH) decrease in RBC. Cells were treated with 40 μM HgCl_2_ for 4 h in the presence of increasing concentrations of *Feijoa* peel or pulp. Data are the means ± SEM (*n* = 9). Statistical significance was calculated with one-way ANOVA followed by Tukey’s test. * (*p* < 0.05) and ** (*p* < 0.01) indicate significant differences from cells lacking *Feijoa* extract treatment.

GSH depletion is a key mechanism of Hg toxicity due to the weakening of the antioxidant defense system. We therefore evaluated the possible protective effect of *Feijoa* peel and pulp extracts on this specific, Hg-induced metabolic condition. As shown in Figure 5, 4 h treatments of RBC with 40 μM HgCl_2_ reduce GSH levels by about 40%. Co-incubation with 20, 80, or 100 μg/mL of *Feijoa* peel extract prevented GSH depletion by about 20% with each concentration, such that GSH levels were unchanged from healthy control levels at the latter two peel extract concentrations. For the pulp extract, data indicate that 20 μg/mL had no effect on GSH levels, while significant protection was observed at 80 and 100 μg/mL, to a maximum of about 90% GSH levels compared to controls.

**Figure 6:**
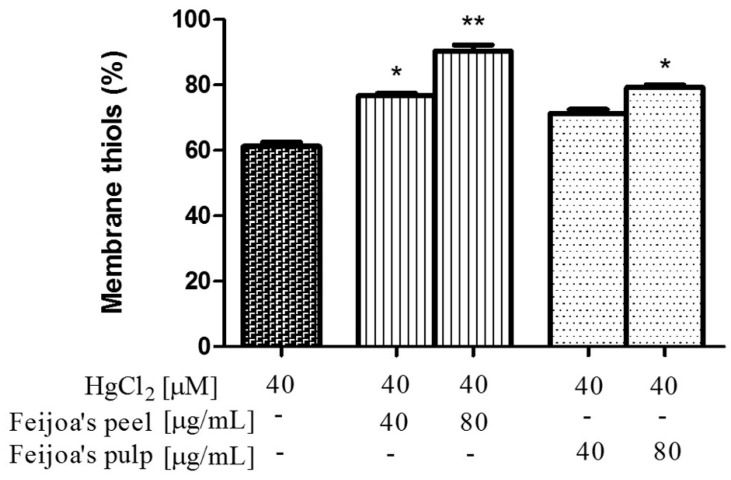
*Feijoa* peel and pulp extracts protect against Hg-induced membrane thiol depletion in RBC. Cells were treated with 40 μM HgCl_2_ for 4 h in the presence of increasing concentrations of *Feijoa* peel or pulp. Data are the means ± SEM (*n* = 9). Statistical significance was calculated with one-way ANOVA followed by Tukey’s test. * (*p* < 0.05) and ** (*p* < 0.01) indicate significant differences from cells lacking *Feijoa* extract treatment.

Based on these results, it seemed appropriate to evaluate the efficacy of peel and pulp extracts in reducing the Hg-induced depletion of membrane thiols, using membranes obtained from intact RBC after incubation with HgCl_2_ (Figure 6). Exposure to 40 µM HgCl_2_ reduced the level of membrane thiols by about 40%. This depletion was significantly counteracted by about 45% and 75% given co-incubation with 40 and 80 µg/mL of peel extract, respectively. Again, the pulp extract was less protective than the peel at the same concentrations, such that membrane thiol depletion was counteracted only at 80 µg/mL, by about 50%.

### 3.4. Peel and Pulp Extracts of Feijoa Reduce Microvesicles Released from Red Blood Cells

**Figure 7:**
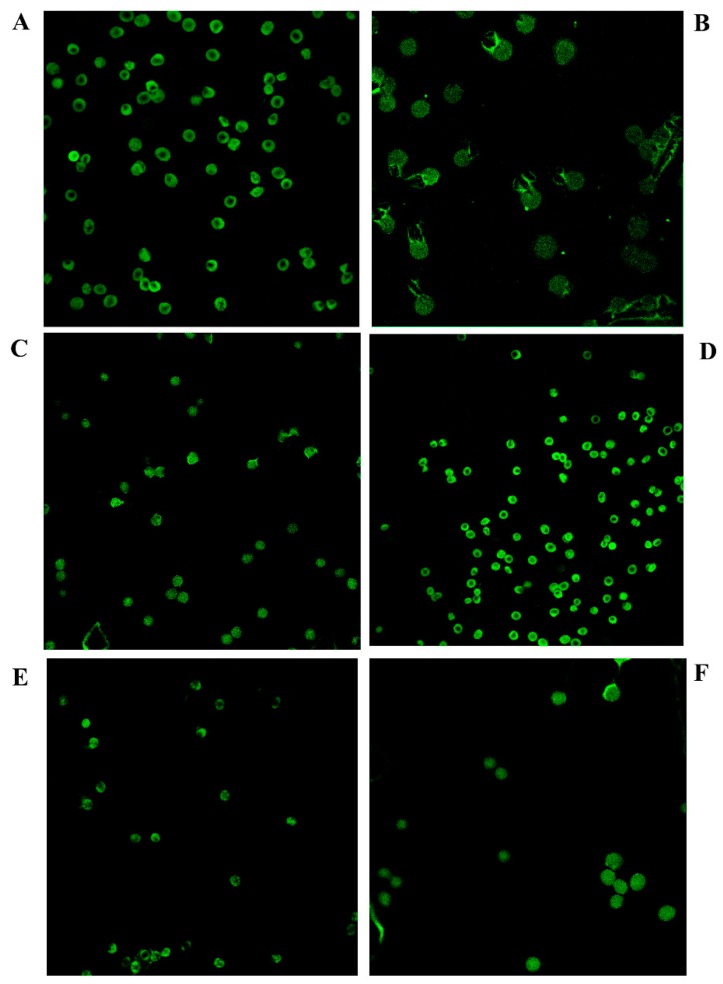
Peel and pulp extracts of *Feijoa* reduce microvesicles (MV) released from RBC. Untreated cells are shown in (**A**). Cells were treated with 40 μM HgCl_2_ for 4 h (**B**) and concurrently treated with 20 or 80 µg/mL of *Feijoa* peel (**C** and **D**, respectively) and pulp (**E** and **F**, respectively) extracts. RBC were stained with Annexin V-FITC.

To investigate the protective role of *Feijoa* extracts on erythrocyte morphological changes and MV formation known to be induced by Hg treatment [41,46], cells treated with HgCl_2_ and peel or pulp extracts, as described in the Materials and Methods section, were analyzed with confocal microscopy. Hg treatment was associated with loss of the typical erythrocyte biconcave shape, as well as the formation of MV clearly discernible on cell membranes (not observable in the control) (Figure 7, Panel A). Cell treatment with *Feijoa* extracts completely restored the typical biconcave shape at 20 µg/mL and 80 µg/mL for peel and pulp, respectively (Figure 7C–F).

## 4. Discussion

Mercury is not only highly toxic, but is an increasingly pervasive dietary heavy metal. As a matter of fact, concerns on the effect of Hg exposure on human health are not only limited to occupationally exposed workers, but also to the general population, mainly via contaminated food ingestion. Although in some European populations the overall Hg daily intake is below the tolerable amount [50,51], appreciable proportions of large fish populations are reported to contain levels of this heavy metal exceeding this amount, up to 2.22 mg/kg wet weight, including anglerfish (*Lophius piscatorius*) and black-bellied angler (*Lophius budegassa*) [52]. Discovering analogous means to simultaneously combat and protect against diet-based Hg toxicity is therefore crucial to public health. In this respect, phytochemicals able to counteract structural and metabolic alterations associated with heavy metal exposure are attractive for the reduction of their toxicity [53,54,55,56]. Data from our group indicate that hydroxytyrosol, an olive oil-derived phenolic antioxidant, has the potential to modulate the toxic effects exerted by Hg in human RBC [41,42,43].

To expand data on the potential role of nutrition in heavy metal toxicity, intact human RBC were exposed to 40 µM HgCl_2_, in line with our previous studies. Several markers of cellular toxicity were then evaluated to test the protective effect of *Feijoa* fruit extracts. According to data reported in similar experimental conditions, RBC treatment with 40 µM HgCl_2_ for 4 h results in a doubling of ROS production, as indicated by DCF fluorescence [41]. Hg-induced ROS generation follows a significant decrease of GSH, which builds up a pro-oxidative microenvironment and renders cells more susceptible to ROS-mediated oxidative damage. A significant decrease in membrane thiols is also detectable in Hg-treated cells. The resulting hemolysis is significantly increased and measurable later, at 24 h.

Here we show the first evidence of *Feijoa* fruit extract protection against HgCl_2_-induced toxic effects in human RBC. The acetonic extracts of both the pulp and peel were able to counteract oxidative stress and cellular thiol decrease in Hg-treated RBC. The peel extract had a greater protective effect compared to the pulp, although to varying extents for the different markers analyzed, which is at least partially due to the greater proportion and diversity of polyphenols in the peel [3,57,58]. Interestingly, the protective effect of the peel from ROS production is only two-fold, compared to an eight-fold effect against overall cytotoxicity indicated by hemolysis. Whereas Hg sequesters and inactivates GSH by binding to sulfhydryl groups, polyphenols act on the resulting ROS by virtue of their hydrogen and electron transfer abilities. The presence of additional bioactive compounds with different activities in the peel (i.e., chelating properties) can also be hypothesized. Remarkably, as little as 10 µg/mL of *Feijoa* peel extract significantly affects all the tested markers, and 80 µg/mL completely prevents Hg-induced ROS production.

The data presented in this paper, although obtained from in vitro studies on human cells, also offer significant experimental evidence that *Feijoa* extracts prevent Hg-induced RBC shape alteration in RBC, which could be taken into account for future clinical investigations. In fact, although a particularly high GSH concentration may partially protect RBC from Hg’s toxic effects, chronic exposure could affect RBC viability and induce morphological changes, also affecting cardiovascular disease. As mentioned before, Hg exposure enhances pro-coagulant activity of these cells, resulting in a contributing factor for Hg-related thrombotic disease [46]. In our previous studies, we raised the fascinating hypothesis that metabolic and shape modification of RBC may be regarded as a clinical biomarker, indicating increased cardiovascular risk in Hg-exposed individuals [41,42].

Our findings, in agreement with the literature data, strengthen the nutritional relevance of *Feijoa* bioactive compounds to the claimed health-promoting effects of this fruit. There is growing interest in utilizing *Feijoa* fruit for human consumption, due to its appetizing quality and its claimed health benefits. *Feijoa* fruit is an excellent source of vitamins and nonessential nutrients, as well as a variety of bioactive compounds endowed with significant antioxidant, antibacterial, and anti-inflammatory activities [10,11,12,13]. In this respect, there is a general agreement that the health-promoting effects of fruit and vegetable intake result from the combined properties and synergistic action of all bioactive constituents, including polyphenols [59,60]. These compounds can improve health due to their strong antioxidant activity, counteracting oxidative stress-induced cellular dysfunctions and modulating key mechanisms implicated in the development of oxidative stress-related human pathologies. Polyphenols are very useful in combating the deleterious effects of heavy metals. For example, Sobeh et al. [61] isolated and identified two compounds from the leaves of *Syzygium samarangense* (myricitrin and 3,5-di-O-methyl gossypetin), both showing antioxidant activities [62,63] and strongly reducing intracellular ROS accumulation and carbonyl content, while also protecting the intercellular GSH levels in keratinocytes (HaCaT) after exposure to sodium arsenite, one of the more toxic environmental heavy metals [61].

*Feijoa* has been proposed as an ideal candidate for nutraceutical strategies in the development of functional foods [64]. The data reported in this paper expands upon the known beneficial effects of *Feijoa* fruit, particularly related to chronic human exposure to heavy metal. In this respect, an interesting observation is that the very low active concentrations utilized in our study could be approached in vivo upon daily intake of *Feijoa* fruit. In this respect, some studies indicate that *Feijoa* fruit extracts are well tolerated in animal models. Karami et al. [65] demonstrated the hepatoprotective activity of methanolic extract of *Feijoa* fruit in a concentration range of 10–100 mg/kg, using the isolated rat liver perfusion system. The same group also investigated nephroprotective effects of leaf extracts (10–40 mg/kg) on renal injury induced by acute doses of ecstasy (MDMA) in mice [66]. Moreover, in a recent study, *Feijoa* leaf extract was shown to be devoid of toxicity in rats up to 2 g/Kg, [11]. Finally, we have confirmed by MTT test, on human leucocytes as well (data not shown), that treatment for 24 h with 5, 50, and 500 μg/mL of acetonic extracts of *F. sellowiana* did not induce significant cytotoxic effects, as already demonstrated on either the Caco-2 or HT-29 cell lines [16]. 

The food industry is increasingly interested in the utilization of non-edible parts of fruits. Phytochemicals are proposed for designing foods with added functional value, aiming to beneficially affect target functions in the body and reduce the risk of diseases. These compounds are present in large quantities in waste products from the agri-food supply chain, especially peels and seeds. Our data, showing a greater protective effect from the *Feijoa* peel on Hg cytotoxicity than from the pulp, corroborates this rationale. Recovering and using such a waste product, normally destined to magnify industrial waste production, would give new life to the less noble part of the fruit. This is further in line with recent studies that propose the potential utilization of *Feijoa* fruit peel for added processing and functional value. As demonstrated by Sun-Waterhouse et al. [64], the extracts produced from *Feijoa* waste, such as the peel, retain high pectin content, which is advantageous for food applications. Moreover, the possibility to utilize *Feijoa* peel-containing food packaging film for the inhibition of foodborne bacteria was recently demonstrated [67].

In conclusion, the novel beneficial properties of *Feijoa* reported in this paper, regarding its efficacy to reduce heavy metal toxicity in human RBC, provide biochemical bases for the use of *Feijoa*-based functional foods or pharmacological preparations in preventing and combating mercury-related illnesses.
